# Racial Differences in Esophageal Squamous Cell Carcinoma: Incidence and Molecular Features

**DOI:** 10.1155/2017/1204082

**Published:** 2017-03-14

**Authors:** Shirui Chen, Kai Zhou, Liguang Yang, Guohui Ding, Hong Li

**Affiliations:** ^1^Shanghai High School, Shanghai 200231, China; ^2^Children's Hospital of Nanjing Medical University, Nanjing 211166, China; ^3^Key Lab of Computational Biology, CAS-MPG Partner Institute for Computational Biology, Shanghai Institutes for Biological Sciences, Chinese Academy of Sciences, Shanghai 200031, China

## Abstract

The incidence and histological type of esophageal cancer are highly variable depending on geographic location and race/ethnicity. Here we want to determine if racial difference exists in the molecular features of esophageal cancer. We firstly confirmed that the incidence rate of esophagus adenocarcinoma (EA) was higher in Whites than in Asians and Blacks, while the incidence of esophageal squamous cell carcinoma (ESCC) was highest in Asians. Then we compared the genome-wide somatic mutations, methylation, and gene expression to identify differential genes by race. The mutation frequencies of some genes in the same pathway showed opposite difference between Asian and White patients, but their functional effects to the pathway may be consistent. The global patterns of methylation and expression were similar, which reflected the common characteristics of ESCC tumors from different populations. A small number of genes had significant differences between Asians and Whites. More interesting, the racial differences of COL11A1 were consistent across multiple molecular levels, with higher mutation frequency, higher methylation, and lower expression in White patients. This indicated that COL11A1 might play important roles in ESCC, especially in White population. Additional studies are needed to further explore their functions in esophageal cancer.

## 1. Introduction

The World Health Organization reported that esophageal cancer was the eighth most common cancer and the sixth cause of cancer-related death in the world [1]. It makes up about 9.3% of all new cancer cases in China, while it makes only 1.0% in the United States [2]. Esophageal cancer includes two major subtypes: esophageal squamous cell carcinoma (ESCC) and esophagus adenocarcinoma (EA). ESCC is more common in developing countries and accounts for more than 90% in Chinese esophageal cancer patients. EA is more common in developed countries and accounts for 80% in United States [1]. Although there are racial differences in the subtypes of esophageal cancer, the comparative studies of molecular biology in different racial groups are lacked.

The recent epidemiological and clinical studies indicate that race/ethnicity is associated with the differences in cancer incidence and mortality [3]. Using a multivariable model adjusted for tumor characteristics and patient-related factors, Warner et al. found Blacks had 21% higher risk of breast cancer-specific death [4]. Similar racial differences were observed for the survival of other cancer types [5, 6]. Furthermore, there exist racial/ethnic differences in cancer-related pathways, such as cell cycle, apoptosis, and proliferation [3]. More and more scientists started to explain the racial difference at the molecular level. A copy number study revealed that 16p gains (East Asian) and 19p losses (Western European) are ethnic-specific chromosomal aberrations in lung adenocarcinoma [7]. Genome sequencing of liver cancer found that AXIN1 was more frequently mutated in patients with HBV infection, while mutations of its downstream gene CTNNB1 majorly occurred in HCV-associated cases [8, 9]. Racial diversity of endometrial cancers [10] and non-small-cell lung cancer [11] were investigated by comparing somatic mutations in common cancer genes. Their results suggested that the mutation frequencies of some genes vary according to race, which could benefit cancer diagnosis and treatment. Except cancer genomes, racial specificity was also observed in other molecular features, such as gene expression in pediatric B-Precursor acute lymphoblastic leukemia [12] and methylation in prostate cancer [13]. However, these studies are still limited to assess the full impact of race in human carcinogenesis.

Here we systematically evaluated the racial differences of esophageal squamous cell carcinoma by integrating epidemiological and molecular data. We found that the global profiles of mutation, methylation, and gene expression were similar between Asian and White patients. The small differences in molecular biology were discussed to understand the impact of race in esophageal squamous cell carcinoma.

## 2. Methods

### 2.1. Data Source

Incidence data of esophageal cancer were collected from Surveillance, Epidemiology, and End Results (SEER) database and two public studies. SEER provides cancer incidence and population data in the United States. We used the SEER*∗*Stat software to calculate age-adjusted incidence rate (during 1992–2012) from SEER-13 registries. Incidence rate in Japan was obtained from a literature that reported data from 15 population-based cancer registries during 1993–2001 by the Japan Cancer Surveillance Research Group [14]. Incidence in China was obtained from another literature that reported cancer incidence and mortality data of 2009 from 104 cancer registries by Chinese National Central Cancer Registry [15]. Due to the low socioeconomic status in China rural areas, we only used incidence data of China urban to reduce the difference of socioeconomic status.

Clinical characteristics and molecular profiles of esophageal cancer patients were obtained from Broad GDAC Firehose. It provided TCGA Level 3 data, including somatic mutations, mRNA expression, and DNA methylation. Due to the relatively low proportions of tumor harboring mutations, we additionally collected somatic mutation data from other genome studies to increase the sample size [16–19]. Totally, we obtained 529 ESCC patients (487 Asians and 42 Whites). Potential functionally effects of somatic mutations were annotated by ANNOVAR [20], including nonsynonymous SNVs, splicing SNVs, stop-gain SNVs, stop-loss SNVs, frame-shift indels, and in-frame indels. Genes were regarded as significantly mutated if their mutation frequencies were higher than 2% and mutation rates were larger than 2 per 1000 bp.

### 2.2. Racial Differences

Our study of racial differences focused on White and Asian population, whose cancer incidence rate and molecular datasets were available. Fisher's exact test was used to compare cancer incidence rate, clinical characteristics, and mutation frequency between Whites and Asians. Differences were considered to be statistically significant at *P* < 0.05.

DNA methylation and gene expression might be affected by clinical characteristics. Therefore, multivariate analysis of variance (ANOVA) was used to find differential genes by race. Clinical features such as race, gender, age, disease stage, and the number of pack-years of smoking were regarded as covariant. To decrease the false-discovery rate in multiple testing, we used a threshold of *P* < 0.05 and fold change (FC) > 2 to select statistically significant change in DNA methylation or gene expression.

All statistical analysis was performed in the software R 3.3.1.

## 3. Results

### 3.1. Racial Difference in the Incidence of Esophageal Cancer Subtypes

**Figure 1:**
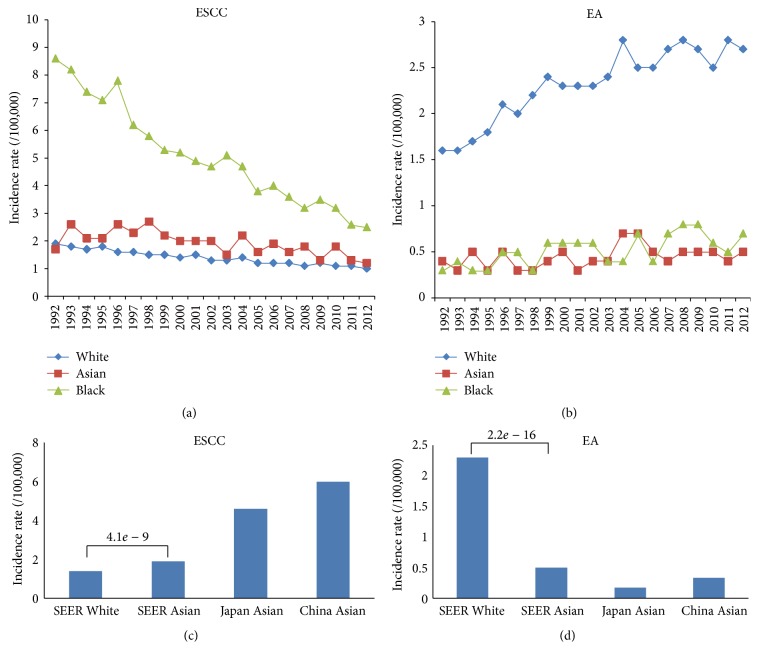
Age-adjusted incidence rate of esophageal cancer by race. (a) and (b) are the incidence of esophageal squamous cell carcinoma (ESCC) and esophagus adenocarcinoma (EA) from 1992 to 2012. Three major races (White, Asian, and Black) were compared to investigate racial difference. (c) and (d) are the comparison of the esophageal cancer incidence among “SEER White,” “SEER Asian,” “Japan Asian,” and “China Asian”. Data of “Japan Asian” and “China Asian” were obtained from cancer surveillance programs in Japan [14] and China [15].

We downloaded SEER datasets and calculated the age-adjusted incidence rate of esophageal cancer. Two esophageal cancer subtypes (ESCC and EA) and three major population (White, Asian, and Black) were analyzed, respectively. The incidence curves of ESCC and EA in 1992–2012 were illustrated in Figures 1(a) and 1(b). The median incidence rates of ESCC among Whites, Asians, and Blacks were 1.4, 1.9, and 4.9 per 100,000, respectively. ESCC incidence rate was highest among Blacks, although it dramatically decreased in recent years. Asians had higher incidence rate of ESCC compared with Whites, and this trend was constant over the 20-year period. The median incidence rates of EA among Blacks, Asians, and Whites were 0.5, 0.5, and 2.3 per 100,000, respectively. The EA incidence rate was highest in White people, and it approximately increased twice in 20 years.

Since SEER collected cancer patients in the United States, the number of Asians and Blacks was smaller than Whites. The smaller population might result in the larger standard error when estimating incidence rate. Therefore, we also used the incidence rate of esophageal cancers in other countries. We excluded Black patients due to the lack of large-scale epidemiological studies. The incidence of ESCC was 4.9 in Japan and 5.9 in China Urban (Figure 1(c)). It further proved that ESCC incidence was higher in Asians than in Whites. On the other hand, the EA incidence rate was higher in White people than in Asian people, no matter the data source (Figure 1(d)). Our subsequent analysis focused on the squamous cell carcinoma (ESCC), which accounts for about 90% of esophageal cancer cases worldwide [21].

### 3.2. ESCC Patients with Genome-Wide Multiomics Data

There were 45 Asian, 42 White, and 4 Black ESCC patients in the TCGA dataset, whose somatic mutation, DNA methylation, and gene expression profiles were available. We excluded Black patients due to the small sample size and only compared the difference between Asians and Whites.  Table 1 summarized the clinical characteristics of ESCC patients by race. The proportion of male in Asian patients (93%) was larger than that for White patients (74%). The distribution of neoplasm stage was significantly different. State I tumors was found in 17% of Whites compared with 0% of Asians. Stage II tumors was found in 40% Whites compared with 76% of Asians. Other clinical characteristics were not significantly different by race.

### 3.3. Racial Difference in Somatic Mutation Landscape

**Figure 2:**
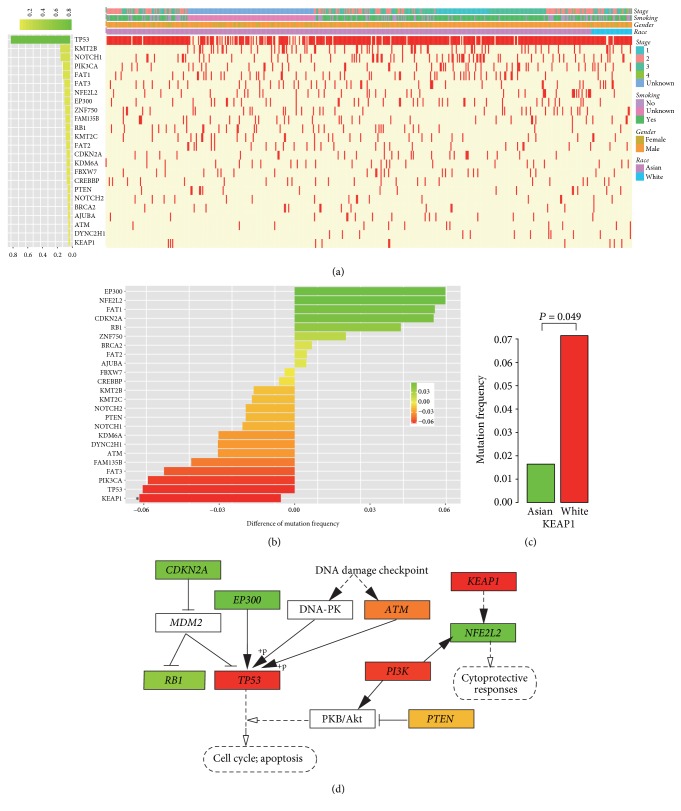
Racial difference of somatic mutations in esophageal squamous cell carcinoma. (a) Mutation landscape of ESCC driver genes in 487 Asian and 42 White patients. (b) Difference of mutation frequencies between Asian and White patients. Red or green indicates higher mutation frequency in Whites or Asians, respectively. (c) Significantly racial difference in KEAP1 mutation frequency. (d) Racial difference in ESCC related pathways. *∗* indicates that the mutation frequency of this gene is significantly different between Asian and White patients.

Cancer is a disease of genome alterations that arise through several driver gene mutations and subsequent passenger mutations [22]. We analyzed the mutation landscape of 529 ESCC patients to selected frequently mutated genes and combined them with previous reported driver genes. We obtained 24 significantly mutated driver genes (SMGs); their mutation spectrum was shown in Figure 2(a). The majority of mutations were found in TP53 (80.1%); other SMGs included KMT2B (13.2%), NOTCH1 (12.8%), and PIK3CA (9.6%). We compared the frequency of gene mutations in Asian and White patients (Figure 2(b)). The overall differences of driver genes were small; only KEAP1 was significantly different. Tumors from White patients were more significantly associated with harboring a KEAP1 mutation (Figure 2(c)).

The 24 SMGs were significantly enriched in pathway in cancer, cell cycle, P53 signaling pathway, JAK-STAT signaling pathway, and Notch signaling pathway. These pathways were further explored by aggregating the racial difference of each driver gene in a pathway. Interesting, some genes belonging to the same pathway showed opposite difference between Asian and White patients (Figure 2(d)). For example, TP53 mutations were more frequent in White patients, while the mutation frequencies of its upstream genes EP300 and CDKN2A were higher in Asian patients. Another example is the Keap1-Nrf2 pathway and White patients harbored more KEAP1 mutations, while Asian people had more NFE2L2 (also called NRF2) mutations. Keap1-Nrf2 pathway is the major regulator of cytoprotective responses to oxidative and xenobiotic damage. Somatic mutations in NRF2 or KEAP1 disrupt the interaction of these two proteins and activate the NRF2 signaling [23]. These results indicated that pathway alterations in ESCC patients were consistent although some genes were more frequently mutated in one population.

### 3.4. Racial Difference in DNA Methylation

**Figure 3:**
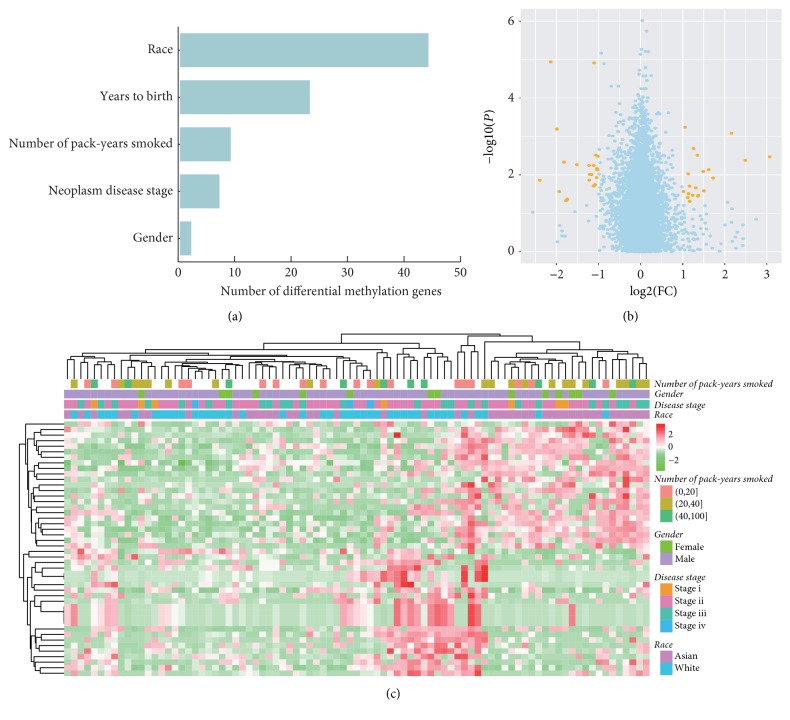
Racial difference of DNA methylation in esophageal squamous cell carcinoma. (a) Number of genes whose methylations were significantly associated with race or other clinical variables. Statistical significance was defined as FC > 2 and *P* < 0.05. (b) Volcano plot (log⁡2 fold change versus log⁡10*P* value) of differences in DNA methylation between Asian and White patients. Significant genes were colored with orange. (c) Heatmap of significantly differential DNA methylation between Asian and White patients.

DNA methylation is a key process in regulating gene expression, which plays important roles in neoplastic development [24]. To identify patients' features associated with DNA methylation, ANOVA was used to correlate methylation values with race, gender, age, disease stage, and the number of pack-years of smoking. Compared to other clinical features, methylation was mostly related to race (Figure 3(a)). Methylation levels of most genes were similar between Asian and White patients (Figure 3(b)), which reflected the common methylation pattern of ESCC tumors. The methylation of 44 genes was significantly associated with race (Figure 3(c)), but they did not significantly enrich functional terms. One interesting gene was the tissue factor pathway inhibitor TFPI, whose methylation was higher in Asians than Whites (*P* = 1.15*E* − 5; FC = 4.35). A similar gene TFPI2 was reported to be frequently methylated in esophageal cancer with a progression tendency [25], which indicated that TFPI methylation might also play roles in esophageal cancer. Another interesting gene was PIK3R1, whose methylation was higher in Asians than in Whites (*P* = 0.01; FC = 2.33). PIK3R1 encodes the 85 kD regulatory subunit of the PI3K heterodimer. Its hypermethylation may relate with lower expression and possibly lead to PI3K pathway activation [26].

### 3.5. Racial Difference in Gene Expression

**Figure 4:**
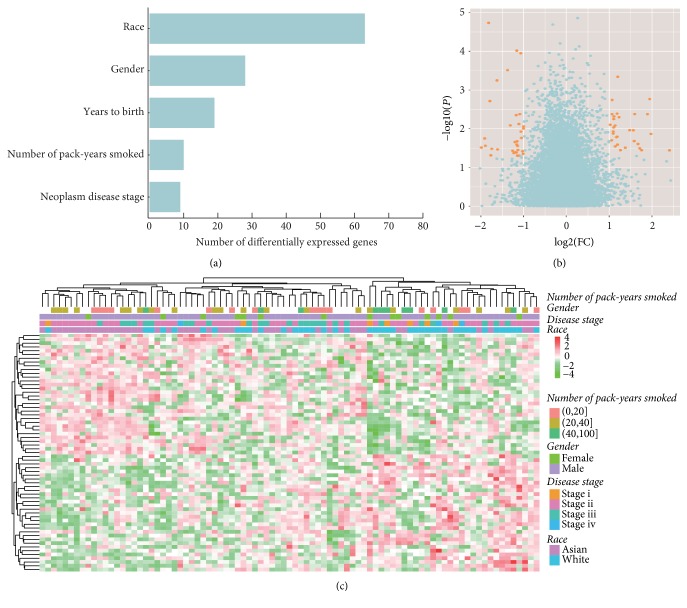
Racial difference of gene expression in esophageal squamous cell carcinoma. (a) Number of genes whose expressions were significantly associated with race or other clinical variables. Statistical significance was defined as FC > 2 and *P* < 0.05. (b) Volcano plot (log⁡2 fold change versus log⁡10* P* value) of differences in gene expression between Asian and White patients. Significant genes were colored with orange. (c) Heatmap of significantly differential gene expression between Asian and White patients.

We analyzed gene expression using the same methods. Similar to DNA methylation, race was the major factor that related to gene expression (Figure 4(a)). The global level of gene expressions was almost unchanged between Asian and White patients (Figure 4(b)). We identified 63 significantly differentially expressed genes (Figure 4(c)). They were enriched in extracellular region, signal peptide, and cell adhesion. The cell adhesion genes may be involved in the tumor microenvironment and cancer progression [27]. Two genes in Wnt signaling pathway were expressed higher in Asians: WNT3 (*P* = 0.03; FC = 2.04) and FZD9 (*P* = 0.004; FC = 2.27). Further studies are needed to explore whether these differences are important to the tumorigenesis of ESCC.

### 3.6. Racial Difference of COL11A1 at Multiple Molecular Levels

**Figure 5:**
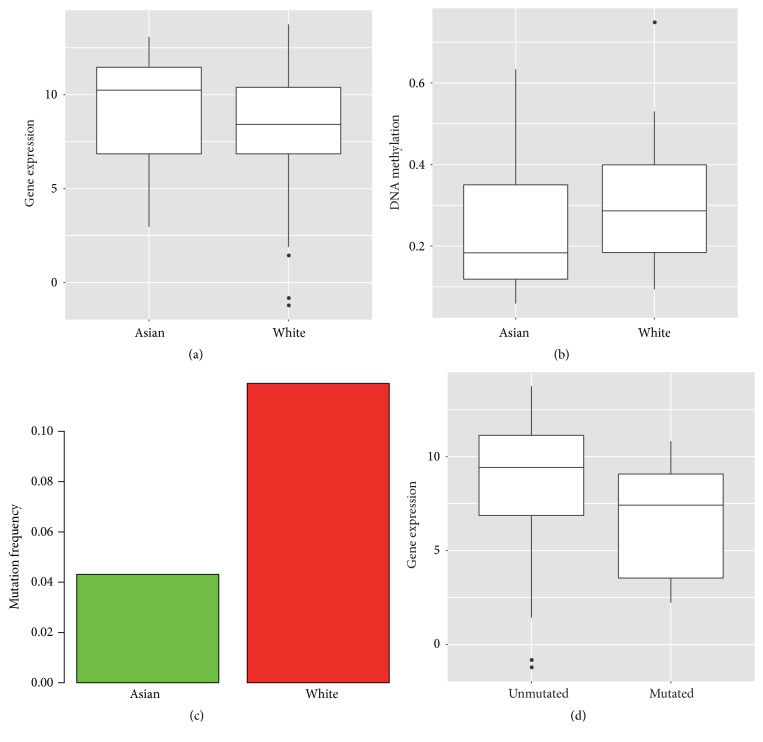
Racial difference of COL11A1 at multiple molecular levels. (a) Gene expression (*P* = 0.01; FC = 3.31). (b) DNA methylation (*P* = 0.003, FC = 0.63). (c) Frequency of somatic mutations (*P* = 0.04). (d) Association between gene mutation and expression (*P* = 0.03).

Finally, we integrated the results of racial difference in mutation, methylation, and expression. COL11A1 was the only gene that was significantly changed in both expression (*P* = 0.01; FC = 3.31) and methylation (*P* = 0.003; FC = 0.63, Figures 5(a) and 5(b)). Its mutation frequency was also significantly different between Asians (4.3%) and Whites (11.9%) (*P* = 0.04, Figure 5(c)). Mutated COL11A1 had higher expression than the wide-type gene (*P* = 0.03, Figure 5(d)). In summary, COL11A1 had higher mutation frequency and higher methylation level in White people, which may decrease gene expression. The consistency of these racial differences across multiple molecular levels is particularly striking.

COL11A1 encodes one of the two alpha chains of type XI collagen, which is a key component of the extracellular matrix. A chemoresistance study reported that downregulation of COL11A1 suppressed chemoresistance in epithelial ovarian carcinoma [28]. Many studies had highlighted the importance of COL11A1 in multiple types of cancer, including colorectal, ovarian, breast, head and neck, lung, and brain cancers [29]. COL11A1 was not reported as a driver gene in previous ESCC genome studies. Our results firstly revealed that COL11A1 might play important roles in ESCC, especially in White population. The racial difference in COL11A1 expression might affect treatment effect.

## 4. Discussion

Since different populations vary substantially in socioeconomic status, diet, virus infection, and environment exposure, some scientists debated the value of racial information in cancer research. However, more and more literatures began to consider racial difference in cancers and other diseases. There are abundant evidences that race is associated with differences in cancer incidence rate, survival time, and drug sensitivity. Genome-wide association studies have found many cancer-susceptible genes, some of which are more common in certain racial/ethnic populations than others. Taken the breast cancer-susceptible gene BRCA1 as an example, genetic BRCA1 mutations were significantly more common in Jewish (10.2%) versus non-Jewish (2.0%) cases [30]. Recently, the larger-scale tumor sequencing projects revealed that pattern and frequency of oncogene mutations might vary by race. For example, the mutation rate of EGFR in non-small-cell lung cancer is 16–18% in North Americans and Europeans, 19% in African-Americans, 22% in Indians, 29% in Koreans, 40% in Japanese, and around 50% in Chinese [31]; such variability in EGFR mutation rate led to different effectiveness of tyrosine kinase inhibitors in the given population. Therefore, racial information is becoming more and more important for the understanding of cancer pathogenesis and development of precision medicine.

There are obvious differences in the incidence of esophageal cancer among different racial groups. Asians have higher incidence rate in esophageal squamous cell carcinoma, no matter whether they live in United States, Japan, or China. Beyond histologic type and incidence rate, we hypothesized that there were racial differences in the molecular biology of esophageal tumors. Therefore, we analyzed the genome-wide mutations, methylations, and expression profiles to determine if racial differences exist among different racial groups. Ours results show that the general patterns of molecular data were similar between Asians and Whites, but small differences exist. Some differences might be important for tumorigenesis and personalized treatment, such as KEAP1 mutation, TFPI methylation, and COL11A1 expression.

We noted that our study had limitations in the sample size. Although we collected 487 Asian cases with mutation data, only 45 cases had methylation and expression profiles. The number of White patients (42) was smaller; gene mutation frequency in Whites may change with the growing sample size. Additionally, we used ANOVA analysis to consider multiple variables that may relate to methylation and expression. The statistical method is reasonable, but the smaller sample size may affect its statistic power. Another limitation is the lack of data on control samples. The use of control samples will enable us to screen cancer-related genes. Unfortunately, the current accumulation of molecular data cannot welly resolve these limitations. Our study will make scientists pay more attention to racial difference, and our results will be helpful for the comprehensive understanding of esophageal squamous cell carcinoma.

## Figures and Tables

**Table 1:** Clinical characteristics of TCGA ESCC patients in different racial groups.

	Whites	Asians	*P* value^#^
Years to birth			0.10
Mean	60.9	56	
Range	(44, 90)	(36, 77)	
Neoplasm disease stage			0.00076^*∗*^
Stage i	7 (17%)	0	
Stage ii	17 (40%)	34 (76%)	
Stage iii	16 (38%)	10 (22%)	
Stage iv	2 (5%)	1 (2%)	
Pathology of T stage			0.00083^*∗*^
T1	6 (14%)	0	
T2	8 (19%)	21 (47%)	
T3	25 (60%)	24 (53%)	
T4	3 (7%)	0	
Pathology of N stage			0.63
N0	24 (57%)	29 (64%)	
N1	13 (31%)	12 (27%)	
N2	3 (7%)	3 (7%)	
N3	2 (5%)	0	
Pathology of M stage			0.59
M0	35 (83%)	44 (98%)	
M1	2 (5%)	1 (2%)	
Gender			0.019^*∗*^
Male	31 (74%)	42 (93%)	
Female	11 (26%)	3 (7%)	
Number of pack-years			0.099
Mean	30	21	
Range	(0, 102)	(0, 50)	

^#^ *P* value is obtained by performing Fisher's exact test between Whites and Asians.

^*∗*^Significant difference (*P* value < 0.05).
